# Quantitative imaging of plants: multi-scale data for better plant anatomy

**DOI:** 10.1093/jxb/erx416

**Published:** 2018-01-23

**Authors:** David Legland, Marie-Françoise Devaux, Fabienne Guillon

**Affiliations:** UR1268 Biopolymères, Interactions et Assemblages, INRA, France

**Keywords:** Image analysis, image fusion, image registration, multi-scale imaging, quantitative histology

## Abstract

This article comments on:

Staedler YM, Kreisberger T, Manafzadeh S, Chartier M, Handschuh S, Pamperl S, Sontag S, Paun O, Schönenberger J. 2017. Novel computed tomography-based tools reliably quantify plant reproductive investment. Journal of Experimental Botany 69, 525–535.

**The ongoing development of imaging systems continuously brings novel possibilities for the exploration of plant anatomy at different scales. However, increasing resolution often results in a smaller field of view, limiting the scope for wider conclusions. Staedler *et al.* (2018) got round this problem by making use of 3D images acquired at two different scales to estimate the number of pollen grains within flowers. It is a powerful approach, providing much more information than with a single scale.**

An understanding of the biological functions, development, or evolution of plants requires an accurate description of their anatomy at various scales: the whole organism, its organs, tissues within each organ, cells within a tissue, the cell walls, or the organelles within a cell. Depending on the representative scale of the structures of interest, various image acquisition devices can be employed to investigate their morphology, chemical composition, or spatial organization (Rousseau *et al.*, 2015) (see Box 1).

**Box 1. Multi-scale imaging of wheat grain**

The anatomical structure of plants can be assessed at different scales. At the largest scale, the whole plant can be imaged using digital photography. The structure is commonly quantified by phenotypic feature related to the size (e.g. length, volume, thickness) of the different organs. The spatial localization of the organs (e.g. position of leaves or fruits on the stem) or their organization may also be relevant. When focusing on a specific organ, a common question is how the different compartments are organized relative to each other. Imaging modalities such as tomography (A) or magnetic resonance imaging (MRI) allow for non-destructive investigation of the global organ geometry (e.g. size, shape, curvature) and organization of different compartments (computed tomography image, A, courtesy of C. Girousse, INRA Clermont-Ferrand). The slicing and staining of samples, followed by macroscopy or microscopy imaging, provides more precise information on the spatial organization of the tissues within an organ (B). When the resolution is sufficient, the size and shape of the cells within tissues may also be assessed (C). At a larger magnification, the use of electron microscopy (D) gives access to more detailed information, such as the morphology of cell walls (e.g. thickness, curvature). Using adequate staining or immuno-labelling also makes it possible to identify specific chemicals, and to describe their spatial localization.

**Figure:**
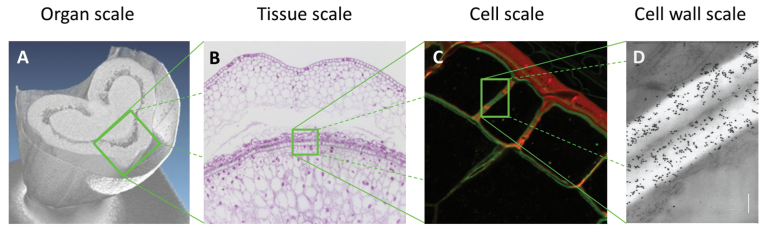

Historically, microscopy has been the usual technique for investigating plant anatomy at the cellular or tissue scale, and the rise of confocal microscopy has allowed us to perceive the 3D structure of tissues or organs with a resolution at the micron level (Truernit *et al.*, 2008). But new technologies – such as the recent development of super-resolution techniques (e.g. PALM or STORM) or the introduction of optical coherence tomography (OCT) (Lee *et al.*, 2006) – continuously bring novel imaging possibilities. For imaging cell walls or organelles within the cells, electron microscopy has often been the method of choice, reaching resolutions at the nanometre scale. The 3D structure can also be assessed, either by combining scanning electron microscopy with serial sectioning of the specimen (Bhawana *et al.*, 2014), or by adapting tomography algorithms to transmission electron microscopy. Magnetic resonance imaging (MRI) and X-ray computed tomography are popular methods for the non-destructive investigation of the 3D architecture of biological specimens, without the need for staining, sectioning or inclusion. The high resolution reached by computed tomography (below the micron) often makes it the best method for the investigation of plant organs (Stuppy *et al.*, 2003; Cloetens *et al.*, 2006; Dhondt *et al.*, 2010; Staedler *et al.*, 2013). Staedler *et al.* (2018) took advantage of this resolution to quantify the 3D anatomy of orchid inflorescences, and through this showed differences in reproductive investment between inflorescences of rewarding and deceiving orchids.

The physical properties of image acquisition devices limit the total quantity of information that can be gained, and so there is a compromise between a high resolution and a large field of view. When the spatial resolution is too low, the smallest structures are difficult to identify. On the other hand, the smaller the field of view, the more difficult it is to cover the totality of the organ of interest with a reasonable acquisition time. The increase in resolution of reconstructed images therefore often corresponds to a reduction in the size of the field of view. This difficulty was encountered in the work of Staedler *et al.* (2018): the structures of interest (pollen grains) could not be imaged with a resolution that allowed their identification while taking into account the whole reference structure (the pollinium, an aggregate of pollen grains). The strategy adopted to circumvent this difficulty was to acquire images at two different resolutions. Images acquired at finer resolution were used for segmentation and counting the pollen grains; images at a coarser resolution were used for assessing the size and shape of the reference structure. The total number of pollen grains was then estimated by combining their numerical density with the volume of the pollinium. It is an approach which exemplifies how data obtained at different resolutions may be used together to provide much more information than data at a single resolution.

The multi-scale investigation of plant tissues is undoubtedly a promising strategy for a better description and understanding of plant anatomy. However, investigation and integration of images, obtained both at different scales and using different imaging modalities (see below), raise new methodological questions (Rousseau *et al.*, 2015).

## Joint exploration of multi-scale images

Investigating plant anatomy at different scales often relies on different imaging modalities. A common approach in microscopy for combining these modalities is correlative microscopy, in particular correlative light and electron microscopy (CLEM) (Bell *et al.*, 2013), and this correlative approach can also be performed with other modalities. For example, the joint analysis of 3D modalities allows the investigation of both anatomy and physiology (Jahnke *et al.*, 2009). Similarly, hyperspectral images obtained from different spectroscopic techniques can be coupled (Allouche *et al.*, 2012). Quantification of the cellular morphology of tomato pericarp was also performed from images obtained using both macroscopy and microscopy imaging, applying a statistical integration approach (Legland *et al.*, 2012). Finally, the multi-scale strategy can be employed for modelling purposes (Mendoza *et al.*, 2007).

In many cases, for example to evaluate the quality of acquisitions, it is of interest to visualize, simultaneously, all the images obtained at different scales on the same sample. Unfortunately, the management and visualization of multiple images obtained with different resolution and/or different orientation still remain complicated. The spatial alignment of two different views of the same object is performed by applying image registration algorithms, which automatically identify the geometric transformation mapping one image onto another (Zitova and Flusser, 2003). Many algorithms have been developed, mostly in the context of medical imaging. The registration of images at similar scales is possible, but is difficult to apply in an automated way when differences in scales are large. Few user-friendly software solutions take into account spatial positioning of images for visualization.

## Fusion of multi-scale quantitative data

Using high-resolution 2D and 3D imaging at the tissue level makes it possible to quantify the 2D and 3D morphology and/or spatial organization of whole biological structures. However, contrary to conventional 2D imaging and except for simple tasks such as counting objects, performing manual measurements on 3D images is challenging – the quantification of anatomical structures therefore strongly relies on adequate image processing and analysis pipelines. The 3D raw images are usually converted into 3D reconstructions of the structures as binary images or geometrical models, and the reconstructed geometry then quantified using adequate descriptors. In the simpler case, structures or structural sections can just be counted. When the structures of interest can be delimited, either manually or by the use of segmentation methods, their geometry can be quantified (e.g. volume, surface area, thickness). When the structure of interest consists of a collection of elements that can be ‘individualized’ (e.g. cells, pores), its geometry can also be described by the shape or size distribution of its elements. In some cases, more complicated descriptors may be envisioned, for example based on skeletization of the microstructure (Mendoza *et al.*, 2007).

Estimating a global quantity from quantifications performed in fields of view with limited size requires a statistical approach. As in Staedler *et al.* (2018), the field of view may be assumed to be representative of the whole organ or tissue under investigation. For some plants, however, tissues may present large variability in morphology depending on their position in the organs. For example, the morphology of cells in fleshy fruit pericarps varies significantly depending on the distance to the outer epidermis. In such cases, an adequate sampling strategy has to be performed, either to integrate the biological variability or to quantify position-dependent variations in morphology.

Another methodological question relates to the differences in resolution of 3D images, which affect the precision and accuracy of measurements taken from them (Arganda-Carreras and Andrey, 2017). When measuring the volume of a biological structure, the estimated values converge when the resolution increases. Increasing the resolution increases the number of details which can be detected and quantified, as well as how many small objects can be distinguished. Contrary to measurement of volume, the measurement of surface area from 3D images thus increases with image resolution, as smaller surface variations are detected. This effect was observed, for example, by Chevallier *et al.* (2014) on food products, with micro-tomography using two different scales. A larger quantity of fine structures (within the overall distribution of structure size) was observed with a smaller voxel size. In plant sciences, a similar effect was observed by Legland *et al.* (2012) in quantifying the morphology of cells in tomato pericarp using two different imaging modalities. The average cell size estimated from macroscopy images was larger than the one obtained after estimation from 3D confocal microscopy (see Box 2).

**Box 2. Quantitative data from plant organs using multi-scale imaging**

Digital images may be processed and analysed to provide quantitative information. However, comparison and integration of quantitative information measured on images obtained at different scales are not always straightforward. In the example shown, images of tomato pericarp have been produced using two acquisition devices. The macroscopy imaging of a pericarp slice allows different tissues to be distinguished (e.g. epidermis, vascular bundles, parenchyma) as well as variations of cell morphology dependent on their location. However, it is difficult to individualize cells due to sampling resolution and the superposition of cell layers.

The cellular morphology within images was quantified using texture analysis tools, measuring variations in shades of grey, and enabling an assessment of mean size variation dependent on the distance to the outer epidermis. A set of 3D confocal laser scanning microscopy images was also acquired along the pericarp and stitched together. The cellular morphology was then quantified by estimating specific cell wall surface area. Assuming that cells are spherical, a typical cell diameter can be estimated, as well as its variation dependent on the distance to the outer epidermis. A comparison of estimated cell diameter obtained from both imaging methods reveals that relative variations are very similar, but that absolute values differ with a scaling factor equal to two or three. The imaging modality at larger scale (here the macroscopy) provides more integrated data, resulting in smoother profiles. The imaging modality with better resolution (here the microscopy) exhibits stronger variability, due to the smaller size of the field of view. The scaling difference between the two profiles can be interpreted as the difference in the resolutions: finer imaging resolves more details, resulting in a smaller estimation of typical cell size.

**Figure:**
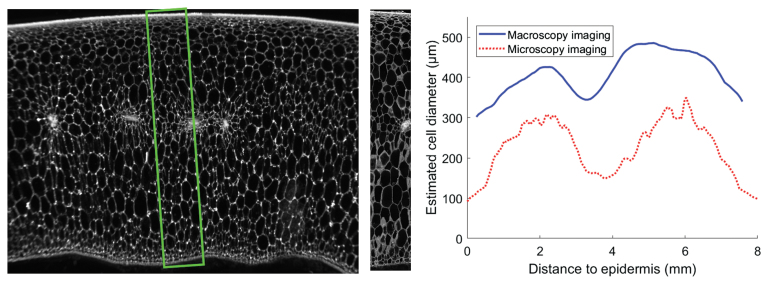

## Computational challenges related to multi-scale imaging

The management of image data obtained at different scales also leads to new computational issues, many of which are reviewed in Walter *et al.* (2010). A first issue is access to data. The large amount of image data, especially when multi-dimensional (3D, time-lapse, multi-channel), is not always well managed by current image-processing software (although specific software and file formats have been developed for accessing high-resolution images produced by slide scanners, using a pyramidal approach). The heterogeneity of specific or proprietary file formats may also restrict accessibility to the data or its reusability.

With the increase in amount of image data, visualization becomes more complicated. Many different projection or rendering methods can be used for exploring multi-dimensional data sets, but the superposition of images obtained from different modalities increases the complexity of the task. In the case of multi- or hyperspectral images, vector spectral data are associated with an image element. Statistical methods such as principal components analyses are therefore necessary to be able to extract relevant information, and to represent its spatial variations within the plant or organ (Geladi and Grahn, 1996).

Quantitative image analysis usually requires the processing of large collections of images to identify relevant factors related to changes in morphology or organization. The proper management of meta-data associated with images obtained on different individuals, with different imaging modalities or at different scales, requires organization and a rigorous approach. Nevertheless, new software solutions such as OMERO (Goldberg *et al.*, 2005) have emerged for the management of large image collections.

To conclude, while modern imaging techniques allow the investigation of plant anatomy at different scales, many challenges still exist for the quantification of data from complex imaging modalities and the fusion of data obtained from different scales, modalities, or datasets.
